# A Multicenter, Randomized, Evaluator-Blinded Study to Examine the Safety and Effectiveness of Hyaluronic Acid Filler in the Correction of Infraorbital Hollows

**DOI:** 10.1093/asj/sjae073

**Published:** 2024-04-04

**Authors:** Brian S Biesman, Jeremy B Green, Rosalyn George, Carolyn Jacob, Melanie Palm, Derek H Jones, Lisa Grunebaum, Kenneth Beer, Young Cho, John H Joseph, Birgitta Almegård, Felipe Weinberg, Torun Bromée

## Abstract

**Background:**

Hyaluronic acid injections are increasingly administered for correction of infraorbital hollows (IOHs).

**Objectives:**

The objective of this study was to examine the effectiveness (IOH correction) and safety of Restylane Eyelight hyaluronic acid (HA_EYE_) injections.

**Methods:**

Patients with moderate/severe IOHs, assessed with the Galderma infraorbital hollows scale (GIHS), were randomized to HA_EYE_ injections (by needle/cannula) (Day 1 + optional Month 1 touch-up) or no-treatment control. The primary endpoint was blinded evaluator–reported Month 3 response, defined as ≥1-point GIHS improvement from baseline (both sides, concurrently). Other endpoints examined investigator-reported aesthetic improvement on the Global Aesthetic Improvement Scale (GAIS), patient-reported satisfaction (FACE-Q satisfaction with outcome; satisfaction questionnaire), and adverse events.

**Results:**

Overall, 333 patients were randomized. Month 3 GIHS responder rate was significantly higher for HA­_EYE_ (87.4%) vs control (17.7%; *P* < .001), and comparable between HA­_EYE_-needle and HA­_EYE_-cannula groups (*P* = .967). HA_EYE_ GAIS responder rate was 87.5-97.7% (Months 3-12). Mean FACE-Q Rasch-transformed scores were 64.3-73.5 (HA_EYE_) vs 14.1-16.2 (control) through Month 12. Patients reported looking younger (≥71%) and less tired (≥79%) with reduced undereye shadows (≥76%) and recovered within 3-5 hours posttreatment. Efficacy was maintained through Month 12 (63.5% GIHS responders) and through Month 18, after Month 12 retreatment (80.3% GIHS responders; 99.4% GAIS responders; FACE-Q scores 72.5-72.8). Forty patients (12.7%) reported typically mild adverse events (4.9% HA_EYE_-needle; 20.9% HA_EYE_-cannula).

**Conclusions:**

HA_EYE_ treatment was effective in correcting moderate/severe IOHs at the primary endpoint (Month 3). Efficacy was sustained through Month 12 after first treatment for 63.5% and through Month 18 for 80.3% (after 1 retreatment) with needle or cannula administration. Safety outcomes were reassuring.

**Level of Evidence: 1:**

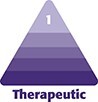

Changes in the infraorbital (tear trough) area of the face can result in “hollowing” around the eye.^1-3^ There is growing interest in hyaluronic acid (HA) injections as an effective nonsurgical approach to correct volume loss and the appearance of infraorbital hollows (IOHs).^3-10^ The tear trough is a notoriously challenging area to treat, requiring precision and an extensive understanding of the changes that affect anatomical structure of the face.^3,9^ Restylane Eyelight HA filler (HA_EYE_; Galderma, Uppsala, Sweden) is formulated with NASHA technology, resulting in a firm/strong gel (high G′) with a low degree of modification and low water absorption ability, making it suitable for this anatomical location.^11,12^ Case series data have shown effectiveness, durability, and high satisfaction with HA_EYE_ in the treatment of IOHs, accompanied by a good safety profile.^10,13-15^ Treatment satisfaction is an important measure of success when assessing nonsurgical aesthetic treatments, particularly when examining therapies that target the infraorbital area, because the eyes are instrumental in projecting emotion as well as influencing perceptions of tiredness and age.^3,16,17^

The current study was a large randomized, controlled investigation to examine effectiveness, safety, and treatment satisfaction following administration of HA_EYE_ treatment for injection in the correction of IOHs. The study aimed to compare treatment outcomes with HA_EYE_ against a no-treatment control among patients with moderate or severe hollowing around the tear trough area.

## METHODS

### Randomized Study Design

A prospective, randomized, evaluator-blinded, no-treatment controlled, parallel group, multicenter study was conducted between November 2019 and April 2022, to evaluate the effectiveness and safety of HA_EYE_ in the correction of IOHs (clinical trial registration no. NCT04154930). The study was carried out in accordance with Good Clinical Practice (GCP) and adhered to the principles of the Declaration of Helsinki. The study protocol was approved by the Food and Drug Administration as well as the Institutional Review Board for each participating study center. The study followed the international standard for clinical study of medical devices for human patients, ISO14155:2011, as applicable, for US regulations, and the International Council for Harmonization of Technical Requirements for Pharmaceuticals for Human Use guideline for GCP (E6) as applicable for medical devices. Patients gave written informed consent. The study objective was to evaluate the effectiveness and safety of HA_EYE_ in the correction of IOHs when administered by needle/cannula injection, with the primary objective of evaluating effectiveness at Month 3 compared with a no-treatment control.

### Study Schedule and Treatment

Assessment scales applied throughout the study are shown in Table 1. Live blinded evaluator assessments were conducted at baseline and then during follow-up visits at Months 3, 6, 9 and 12. Treating investigator assessments were conducted at baseline and then at Months 1, 3, 6, 9 and 12.

**Table 1.** Summary of Assessment Scales

GIHS (Blinded evaluator assessment)	GAIS (Treating investigator assessment)	FACE-Q Psychological function (response scale) (Patient-reported)	Patient satisfaction questionnaire (Patient-reported)
0 (none)	Very much improved	1 (definitely disagree)	Very satisfied
1 (mild)	Much improved	2 (somewhat disagree)	Satisfied
2 (moderate)	Improved	3 (somewhat agree)	Neutral
3 (severe)	No change	4 (definitely agree)	Dissatisfied
	Worse		Very dissatisfied
	Much worse		
	Very much worse		

GAIS, Global Aesthetic Improvement Scale; GIHS, Galderma infraorbital hollow scale.

Patients were randomized 6:1 to receive either HA_EYE_ treatment in the infraorbital area or no treatment (control). For patients randomized to HA_EYE_, the first treatment was administered at baseline with an optional touch-up treatment at Month 1. An additional (optional) retreatment was offered at Month 12 for patients treated with HA_EYE_ at baseline in whom optimal aesthetic improvement had not been maintained. Patients in the no-treatment control group were also offered an HA_EYE_ treatment at Month 12.

HA_EYE_ was supplied in syringes containing 0.5 mL HA_EYE_ (20 mg/mL stabilized HA and 3 mg/mL lidocaine). Approximately half of all study sites were selected to administer HA_EYE_ treatment by needle (HA_EYE_-needle group) and the remaining sites administered treatment by cannula (HA_EYE_-cannula group) for all patients randomized to treatment. The same injection method (needle or cannula) performed at baseline was implemented at each subsequent touch-up treatment. The injection technique was at the discretion of the treating investigator. However, the trial protocol recommended serial puncture for needle injection and the fanning technique for cannula administration. HA_EYE_ was placed in the supraperiosteal plane, at the junction of the lower eyelid and midface where a volume deficit had formed. This part of the face comprises the area bordered by the nasal sidewall medially, the temporal region of the bony orbit laterally, the bulk of the lower eyelid superiorly, and the superior aspect of the midface inferiorly.

All patients who received optional retreatment/treatment at Month 12 were followed up for effectiveness and safety outcomes for another 6 months. Treating investigators performed effectiveness and safety assessments at Months 13, 15 and 18 (ie, 1, 3 and 6 months postretreatment or post first treatment at Month 12 for controls). Blinded evaluator effectiveness assessments were also conducted at Months 15 and 18. A maximum injection volume of 1 mL (per side) was administered for each baseline treatment, touch-up, and optional treatment.

### Study Population

Males and females, age >21 years, who had normal visual function test results were included in the study. Patients were required to have grade 2 (moderate) or 3 (severe) hollowing in the infraorbital area, according to blinded evaluator assessments, graded with the 4-point Galderma infraorbital hollows scale (GIHS): 0 (none), 1 (mild), 2 (moderate), or 3 (severe).^18^

Patients were excluded if they had a known or previous allergy or hypersensitivity to any injectable HAs, any HA_EYE_ constituents, or gram-positive bacterial proteins. Patients were not allowed to enter the study if they had previous or planned facial plastic surgery or cosmetic procedure(s) (eg, laser or chemical resurfacing, needling, facelift, or radiofrequency) that might interfere with effectiveness assessments. Any history of recurrent or chronic infraorbital edema, rosacea, uncontrolled severe seasonal allergies, inflammation, or pigmentation abnormalities around the eye area, retinal disease, detached retina or other condition associated with declining visual acuity precluded inclusion in the study. Patients could not participate if they had demonstrated weight loss or gain (≥2 body mass index [BMI] units) within 90 days of the study or planned to significantly gain or reduce their weight during the study period, or if they were pregnant, planning a pregnancy, or breastfeeding. In addition, patients were excluded if they had undergone previous lower eyelid surgery, including orbital or midface surgery; if they had a permanent implant or fat grafting or fat injections in the midfacial region that could interfere with effectiveness assessments; if they had lower lid retraction or exophthalmos, ectropium, entropion, or trichiasis of the lower eyelid; if they had a tendency to accumulate eyelid edema, had developed festoons, or had large or herniating infraorbital fat pads, skin or fat atrophy other than age-related in the midfacial or periorbicular regions; or if they had been diagnosed with a connective tissue disorder, skin laxity, or sun damage beyond typical for the patient's age.

### Treatment Effectiveness and Patient Satisfaction Assessments

The primary effectiveness endpoint was the responder rate at Month 3, defined as those patients achieving ≥1-point improvement from baseline GIHS score on both sides of the face, concurrently, based upon blinded evaluator live assessment. Secondary effectiveness endpoints included responder rate (blinded evaluator assessments) at Months 6, 9, and 12 posttreatment and at Months 15 and 18 after optional retreatment/treatment (ie, 1, 3 and 6 months following retreatment or post first treatment at Month 12 for controls). Overall aesthetic improvement was evaluated as the proportion of patients achieving a Global Aesthetic Improvement Scale (GAIS) rating of “improved” or above (Table 1), based upon separate treating investigator and patient self-assessment evaluations, at all follow-up visits from baseline through Month 12 and at Months 15 and 18 (equivalent to 3 and 6 months following optional retreatment/treatment at Month 12).

Satisfaction with treatment results was assessed with the FACE-Q satisfaction with outcome questionnaire and patient satisfaction questionnaire. Responses for both questionnaires were reported at Months 1, 3, 6, 9, and 12 from baseline, and at Months 15 and 18 (equivalent to 3 and 6 months following optional retreatment/treatment at Month 12). FACE-Q responses were converted to Rasch-transformed total scores to examine the change in patient satisfaction at each study visit.

Recovery time after treatment was reported according to the time taken for patients to feel comfortable returning to social engagement (eg, workplace, social event), based on patient diary card reporting.

### Assessment of Safety

Adverse events (AEs) were reported throughout the study period by treating investigators. Visual function assessments (including Snellen visual acuity [VA], extraocular muscle function, and confrontation visual field testing) were performed both before and 30 minutes postinjection at baseline, optional touch-up, and optional retreatment/treatment. In addition, visual function assessments were performed on Day 14 after each treatment. Changes in Snellen VA of 1 line or more were reported as AEs of special interest (AESIs). Patients also reported in their diaries any incidence of predefined/expected symptoms (bruising, redness, pain, tenderness, lumps/bumps, itching, or swelling) or any other symptoms occurring during the first 28 days after treatment.

### Statistical Methods

All statistical analyses were conducted with the SAS Version 9.4. Most variables were analyzed and reported according to the injection tool and group: HA_EYE_-needle, HA_EYE_-cannula, HA_EYE_-pooled (all patients receiving HA_EYE_ treatment by needle or cannula) and control (no-treatment).

Randomization of patients to treatment was performed with a computer-generated randomization list stratified by Fitzpatrick skin type (FST) group (I-III, IV, and V-VI) and study site. The FST IV and FST V-VI groups were further stratified at needle or cannula level (needle sites were pooled into a needle stratum, and cannula sites pooled into a cannula stratum). Randomization numbers were allocated in ascending sequential order to each patient.

The intention-to-treat (ITT) population comprised all patients randomized at baseline. Due to the COVID-19 pandemic, some patients had remote visits during the study. Therefore a modified intention-to-treat (mITT) population was created which included all patients in the ITT who did not have a GIHS outcome reported by remote assessment at Month 3. The per protocol population included all patients in the mITT population who completed the Month 3 posttreatment visit without any deviations considered to have a substantial impact on the primary effectiveness. The safety population included all patients who were treated with HA_EYE_ or randomized to the control group and were analyzed according to the as-treated principle.

The primary effectiveness analysis for Month 3 GIHS responder rate vs control was performed for the mITT population with the Cochran-Mantel-Haenszel (CMH) test stratified by injection tool, with a sensitivity analysis conducted with the ITT population. Missing values were handled with multiple imputation. The Breslow-Day test for homogeneity of odds ratios was performed to assess consistency of treatment effect across injection tools. Secondary GIHS responder rates were analyzed with the CMH test stratified by injection tool on the ITT population (observed cases). Confidence intervals were calculated with normal approximation (Wald) throughout. All other endpoints were analyzed descriptively only.

## RESULTS

### Study Population

Demographic and baseline characteristic data are shown in Table 2. Overall, 333 patients at 16 study sites in the US were randomized. Most patients were female (87.1%), White (88.9%), and not of Hispanic or Latino origin (77.5%). Mean (standard deviation [SD]) age was 44.4 (11.67) years (range 22-73 years). The majority had Fitzpatrick skin type III (41.1%) or II (25.8%), and mean BMI was 25.4 kg/m^2^. All patients had GIHS scores of 2 (51.4% right; 52.3% left) or 3 (48.6% right; 47.7% left) at baseline. In total, 287 were randomized to receive HA_EYE_ treatment (HA_EYE_-pooled); 148 were in the HA_EYE-_needle group and 139 in the HA_EYE_-cannula group. The control group (no treatment) contained 46 patients.

**Table 2.** Patient Demographics and Baseline Characteristics (ITT Population)

	HA_EYE_-needle (*n* = 148)		HA_EYE_-cannula (*n* = 139)		HA_EYE_-pooled (*n* = 287)		Control (*n* = 46)		Total (*n* = 333)	
Age (years)										
Mean (SD)	44.3 (12.15)		44.2 (10.98)		44.3 (11.58)		45.5 (12.27)		44.4 (11.67)	
Range	22-73		24-72		22-73		24-63		22-73	
>45 years, *n* (%)	71 (48.0)		69 (49.6)		140 (48.8)		22 (47.8)		162 (48.6)	
Sex, *n* (%)										
Female	130 (87.8)		122 (87.8)		252 (87.8)		38 (82.6)		290 (87.1)	
Male	18 (12.2)		17 (12.2)		35 (12.2)		8 (17.4)		43 (12.9)	
Race, *n* (%)										
White	133 (89.9)		124 (89.2)		257 (89.5)		39 (84.8)		296 (88.9)	
Black/African American	5 (3.4)		12 (8.6)		17 (5.9)		4 (8.7)		21 (6.3)	
Asian	3 (2.0)		1 (0.7)		4 (1.4)		1 (2.2)		5 (1.5)	
Native Hawaiian or other Pacific Islander	0		1 (0.7)		1 (0.3)		0		1 (0.3)	
Other	7 (4.7)		1 (0.7)		8 (2.8)		2 (4.3)		10 (3.0)	
Ethnicity, *n* (%)										
Hispanic or Latino	40 (27.0)		26 (18.7)		66 (23.0)		9 (19.6)		75 (22.5)	
Not Hispanic or Latino	108 (73.0)		113 (81.3)		221 (77.0)		37 (80.4)		258 (77.5)	
Fitzpatrick skin type, *n* (%)										
I–III	97 (65.5)		100 (71.9)		197 (68.6)		31 (67.4)		228 (68.5)	
IV	36 (24.3)		26 (18.7)		62 (21.6)		10 (21.7)		72 (21.6)	
V	9 (6.1)		4 (2.9)		13 (4.5)		3 (6.5)		16 (4.8)	
VI	6 (4.1)		9 (6.5)		15 (5.2)		2 (4.3)		17 (5.1)	
Blinded evaluator GIHS score, *n* (%)	Right	Left	Right	Left	Right	Left	Right	Left	Right	Left
2	79 (53.4)	76 (51.4)	68 (48.9)	71 (51.1)	147 (51.2)	147 (51.2)	24 (52.2)	27 (58.7)	171 (51.4)	174 (52.3)
3	69 (46.6)	72 (48.6)	71 (51.1)	68 (48.9)	140 (48.8)	140 (48.8)	22 (47.8)	19 (41.3)	162 (48.6)	159 (47.7)

A no-treatment control arm was utilized for comparison with HA_EYE_-needle and HA_EYE_-cannula treatment groups throughout the study. The ITT population comprised all patients who were randomized to the study at baseline. HA_EYE_-pooled included all patients receiving HA_EYE_ treatment by needle or cannula. GIHS, Galderma infraorbital hollow scale; HA_EYE_, Restylane Eyelight hyaluronic acid treatment; ITT, intention-to-treat; SD, standard deviation.

Table 3 shows the injection volumes administered at each treatment and touch-up visit. The mean total injection volume, including baseline treatment and touch-up treatments, was similar for patients treated with HA_EYE_ by needle (2.0 mL) and those treated by cannula (2.3 mL). Overall, 164 patients received HA_EYE_ retreatment at Month 12; 88 by needle (mean volume 1.0 mL) and 76 by cannula (mean volume 1.2 mL). All HA_EYE_-needle participants received supraperiosteal injections only, whereas 99.3% of patients who received HA_EYE_-cannula had supraperiosteal injections and 16.7% also received injections at other depths. At the baseline treatment, 73.2% and 26.8% of patients received HA_EYE_-cannula treatment by a 25-gauge and a 27-gauge cannula, respectively. At Month 12, 32 patients in the control group opted to receive HA_EYE_ treatment; 17 were injected by needle (100% supraperiosteal injections only; mean volume 1.5 mL) and 15 by cannula (100% had supraperiosteal injections and 13.3% had other depths; mean volume 1.5 mL). At Month 12, the controls received HA_EYE_-cannula treatment by 25-gauge (80.0%) and 27-gauge (20.0%) cannula. With both needle and cannula, various injection techniques were employed, the most common being serial puncture (as recommended) for needle injections, performed in 59% to 80% of patients across the treatment occasions, and linear retrograde for cannula injections, in 79% to 96% of patients. Around 20% to 33% of patients were injected with the recommended fanning technique for the cannula. A summary of the injection techniques administered at each treatment occasion is presented in Table 4.

**Table 3.** Injection Volumes at Baseline Treatment and Touch-up (Safety Population)

	Injection volume, mL		
	HA_EYE_-needle (*n* = 163)	HA_EYE_-cannula (*n* = 153)	HA_EYE_-pooled (*n* = 316)
Baseline treatment			
*n*	146	138	284
Mean (SD)	1.3 (0.4)	1.4 (0.5)	1.3 (0.5)
Median	1.2	1.3	1.3
Min, max	0.4, 2.0	0.4, 2.0	0.4, 2.0
Optional touch-up			
*n*	113	108	221
Mean (SD)	1.0 (0.5)	1.1 (0.5)	1.0 (0.5)
Median	1.0	1.0	1.0
Min, max	0.2, 2.0	0.2, 2.0	0.2, 2.0
Baseline treatment + touch-up			
*n*	146	138	284
Mean (SD)	2.0 (0.8)	2.3 (0.9)	2.1 (0.9)
Median	2.0	2.0	2.0
Min, max	0.45, 4.0	0.4, 4.0	0.4, 4.0
Optional retreatment			
*n*	88	76	164
Mean (SD)	1.0 (0.5)	1.2 (0.6)	1.1 (0.5)
Median	1.0	1.0	1.0
Min, max	0.2, 2.0	0.3, 2.0	0.2, 2.0
Optional first treatment (no-treatment control)			
*n*	17	15	32
Mean (SD)	1.5 (0.6)	1.5 (0.5)	1.5 (0.5)
Median	1.7	1.5	1.6
Min, max	0.1, 2.0	0.5, 2.0	0.1, 2.0

Baseline treatment, optional touch-up treatment, and optional retreatment all refer to patients randomized to HA_EYE­_ treatment at baseline. Optional baseline treatment only refers to those patients in the no-treatment control group at baseline who were offered HA_EYE_ treatment at Month 12. HA_EYE_-pooled includes all patients receiving HA_EYE_ treatment by needle or cannula. HA_EYE_, Restylane Eyelight hyaluronic acid treatment; max, maximum; min, minimum; SD, standard deviation.

**Table 4.** Injection Techniques at Each Treatment (Safety Population)

	*n* (%)		
	HA_EYE_-needle (*n* = 163)	HA_EYE_-cannula (*n* = 153)	HA_EYE_-pooled (*n* = 316)
Baseline treatment			
*n*	146	138	284
Linear antegrade	20 (13.7)	12 (8.7)	32 (11.3)
Linear retrograde	13 (8.9)	126 (91.3)	139 (48.9)
Fanning	0	46 (33.3)	46 (16.2)
Microbolus	67 (45.9)	18 (13.0)	85 (29.9)
Serial puncture	117 (80.1)	0	117 (41.2)
Optional touch-up			
*n*	113	108	221
Linear antegrade	17 (15.0)	5 (4.6)	22 (10.0)
Linear retrograde	7 (6.2)	104 (96.3)	111 (50.2)
Fanning	0	29 (26.9)	29 (13.1)
Microbolus	49 (43.4)	17 (15.7)	66 (29.9)
Serial puncture	84 (74.3)	0	84 (38.0)
Optional retreatment			
*n*	88	76	164
Linear antegrade	14 (15.9)	12 (15.8)	26 (15.9)
Linear retrograde	14 (15.9)	60 (78.9)	74 (45.1)
Fanning	0	23 (30.3)	23 (14.0)
Microbolus	45 (51.1)	21 (27.6)	66 (40.2)
Serial puncture	60 (68.2)	0	60 (36.6)
Optional first treatment (no-treatment control)			
*n*	17	15	32
Linear antegrade	2 (11.8)	0	2 (6.3)
Linear retrograde	3 (17.6)	14 (93.3)	17 (53.1)
Fanning	0	3 (20.0)	3 (9.4)
Microbolus	10 (58.8)	4 (26.7)	14 (43.8)
Serial puncture	10 (58.8)	0	10 (31.3)

Baseline treatment, optional touch-up treatment, and optional retreatment all refer to patients randomized to HA_EYE­_ treatment at baseline. Optional first treatment only refers to those patients in the no-treatment control group at baseline who were offered HA_EYE_ treatment at Month 12. HA_EYE_-pooled includes all patients receiving HA_EYE_ treatment by needle or cannula. HA_EYE_, Restylane Eyelight hyaluronic acid treatment.

### Effectiveness Outcomes

**Figure 1.**
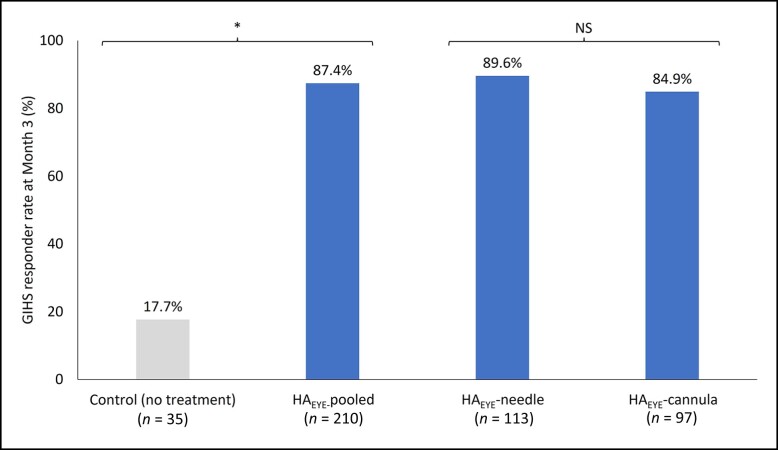
GIHS responder rate at Month 3: blinded evaluator assessment (mITT population). The difference between GIHS responder rates in the HA_EYE_-pooled group and the control group was statistically significant (*P* < .001) at Month 3. The analysis was performed with multiple imputation for missing data and the Cochran-Mantel-Haenszel test stratified by injection tool. The difference in effect of HA_EYE_ when administered by needle or by cannula was not statistically significant (*P* = .967). Breslow-Day test was performed to assess the homogeneity of the odds ratios across injection tools. The mITT population included all patients who were randomized at baseline and who did not have their GIHS Month 3 visit assessment conducted remotely. HA_EYE_-pooled included all patients receiving HA_EYE_ treatment by needle or cannula. GIHS, Galderma infraorbital hollow scale; HA_EYE_, Restylane Eyelight hyaluronic acid treatment; mITT, modified intention-to-treat; NS, not significant.

Figure 1 shows the Month 3 GIHS responder rate based upon blinded evaluator assessment. The GIHS responder rate at Month 3 after first treatment for all HA_EYE_ recipients (87.4%) was statistically significantly higher (*P* < .001) than in the no-treatment control group (17.7%). The responder rate was 89.6% in the needle group and 84.9% in the cannula group. The difference in effect of HA_EYE_ when administered by needle or cannula was not statistically significant (*P* = .967). Results were similar for the per protocol population and other sensitivity analyses.

**Figure 2.**
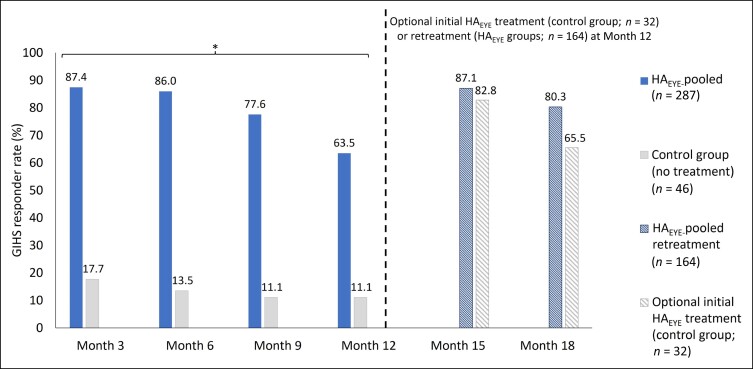
Blinded evaluator–reported GIHS responder rate at Months 6, 9, and 12 and at Months 15 and 18 (3 and 6 months after optional treatment/retreatment at Month 12) (observed cases; ITT population). The difference between GIHS responder rates in the HA_EYE_-pooled group and the control (no-treatment) group was statistically significant at months 6, 9 and 12 (*P* < .001; Cochran-Mantel-Haenszel test stratified by injection tool). An optional HA_EYE_ treatment was offered at Month 12 to patients treated at baseline in whom optimal aesthetic improvement had not been maintained, and to patients in the control group. Only patients who had optional HA_EYE_ retreatment (*n* = 164) or initial treatment at Month 12 (control group; *n* = 32) attended visits at Months 15 and 18 (corresponding to 3 and 6 months after retreatment/treatment). The ITT population included all patients who were randomized at baseline. HA_EYE_-pooled included all patients receiving HA_EYE_ treatment by needle or cannula. GIHS, Galderma infraorbital hollow scale; HA_EYE_, Restylane Eyelight hyaluronic acid treatment; ITT, intention-to-treat.

The GIHS responder rate remained statistically significantly higher in the HA_EYE­_-pooled group compared with the control group at Months 6, 9 and 12 after first treatment (*P* < .001; Figure 2). GIHS responder rates were 86.0% (Month 6), 77.6% (Month 9) and 63.5% (Month 12) in the HA_EYE­_-pooled group. Respective GIHS responder rates in the no-treatment control group were 13.5%, 11.1%, and 11.1%.

HA_EYE_-pooled data showed that patients receiving an optional retreatment at Month 12 demonstrated GIHS responder rates of 87.1% and 80.3% at follow-up visits 3 and 6 months later (Month 15 and Month 18). Patients in the no-treatment control group who received an HA_EYE­_ treatment at Month 12 demonstrated GIHS responder rates of 82.8% and 65.5% at follow-up visits 3 and 6 months later.

**Figure 3.**
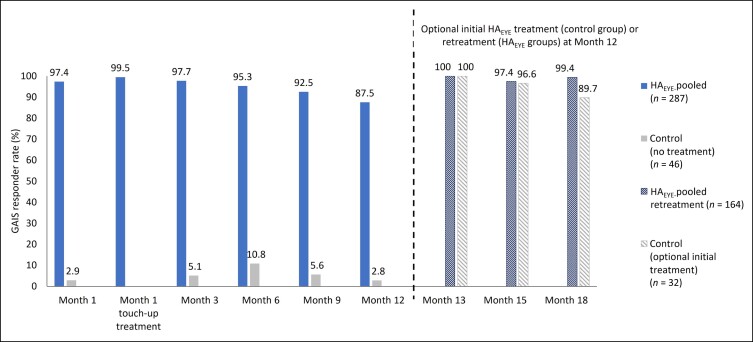
Treating investigator–reported GAIS responder rate at each study visit following HA_EYE_ treatment (observed cases; ITT population). A responder was defined as a patient who indicated that their appearance was “improved,” “much improved,” or “very much improved” on the GAIS. An optional additional HA_EYE_ treatment was offered at Month 12 to patients already treated at baseline in whom optimal aesthetic improvement had not been maintained, and to patients in the control group. Only patients who had HA_EYE_ retreatment/treatment at Month 12 attended visits at Months 13, 15 and 18 (corresponding to 1, 3, and 6 months after retreatment/treatment at Month 12). The ITT population included all patients who were randomized at baseline. HA_EYE_-pooled included all patients receiving HA_EYE_ treatment by needle or cannula. GAIS, Global Aesthetic Improvement Scale; HA_EYE_, Restylane Eyelight hyaluronic acid treatment; ITT, intention-to-treat.

GAIS responder rates according to treating investigator assessments at each study visit are shown in Figure 3. GAIS responder rate for HA_EYE_-pooled patients was 97.4% at Month 1 and rose to 99.5% among those receiving a touch-up treatment. HA_EYE_-pooled data showed that GAIS responder rates were 97.7% at Month 3, 95.3% at Month 6, 92.5% at Month 9, and 87.5% at Month 12 after first treatment. HA_EYE_ injection methods by needle and by cannula resulted in similar responder rates. GAIS responder rates ranged between 2.9% and 10.8% in the control group during the 12-month study period. HA_EYE_ recipients who had an optional retreatment at Month 12 continued to show high GAIS responder rates at Months 13 (100%), 15 (97.4%), and 18 (99.4%). Patients in the control group who chose to receive an HA_EYE_ treatment at Month 12 demonstrated GAIS responder rates of 100% (Month 13), 96.6% (Month 15), and 89.7% (Month 18). Patient-reported GAIS responder rates showed a similar pattern to those assessed by treating investigators throughout the study period (with responder rates of 95.0%, 79.8%, and 93.0% at Months 3, 12, and 18 in the group randomized to HA_EYE_).

**Figure 4.**
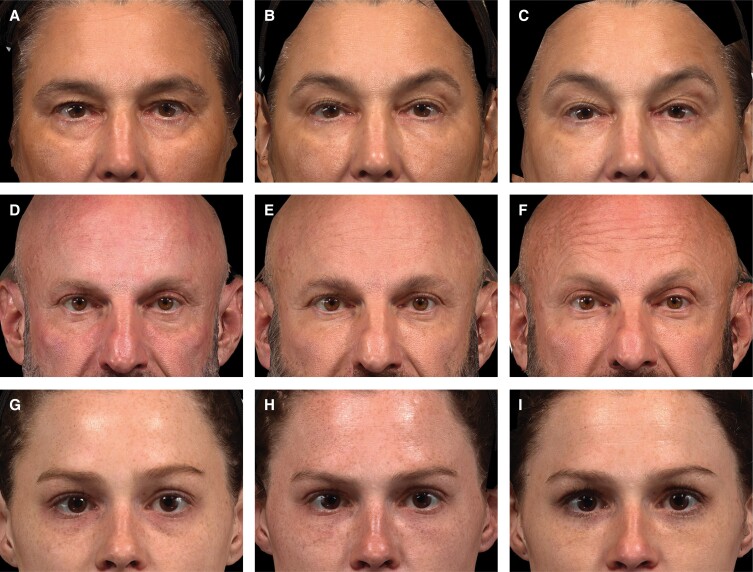
Photographic outcomes for 3 patients before and after treatment with HA_EYE_. Images A-C show a White, 54-year-old, postmenopausal female treated with 2.0 mL HA_EYE_ with a needle (1.0 mL on each side). (A) Before treatment, blinded evaluator GIHS score 3 (severe, both sides); (B) at Month 3, GIHS score 1 (mild, both sides); (C) at Month 12 before retreatment, GIHS score 0 (none, right) and 1 (mild, left). Images D-F show a White, 51-year-old male treated with 4.0 mL HA_EYE_ (2.0 mL on each side) by cannula injection. (D) Before treatment, blinded evaluator GIHS score 3 (severe, both sides); (E) at Month 3, GIHS score 1 (mild, both sides); (F) at Month 12 before retreatment, GIHS scores 2 (moderate, right), 3 (severe, left). Images G-I show a White, 26-year-old female treated with 1.8 mL HA_EYE_ (0.8 mL on right side and 1.0 mL on left side) by needle injection. (G) Before treatment, blinded evaluator GIHS score 2 (moderate, both sides); (H) at Month 3, GIHS score 0 (none, both sides); (I) at Month 12 before retreatment, GIHS score 1 (mild, both sides). GIHS, Galderma infraorbital hollow scale.

Photographic outcomes are provided in Figure 4 for 3 patients, showing the treatment area before HA_EYE_ injections, at Month 3, and at Month 12.

### Satisfaction Outcomes

**Figure 5.**
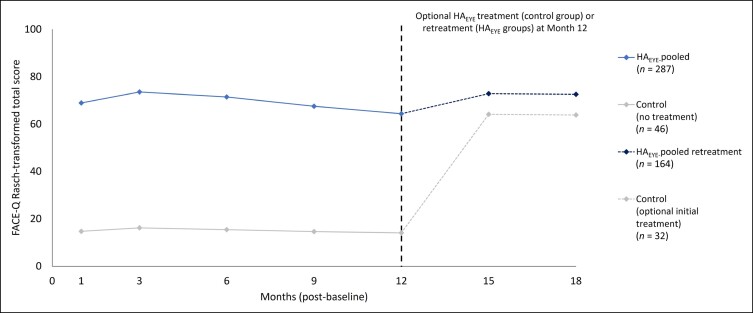
FACE-Q satisfaction with outcome Rasch-transformed mean total scores at each visit (observed cases; ITT population). Satisfaction with outcome Rasch-transformed total scores ranged from 0 (worst) to 100 (best). Higher scores reflect a better outcome. Only patients who received an HA_EYE_ retreatment (*n* = 164) or optional initial treatment (control group; *n* = 32) had visits at Months 15 and 18 (corresponding with 3 and 6 months after retreatment/treatment at Month 12). The ITT population included all patients who were randomized at baseline. HA_EYE_-pooled included all patients receiving HA_EYE_ treatment by needle or cannula. HA_EYE_, Restylane Eyelight hyaluronic acid treatment; ITT, intention-to-treat.

Mean FACE-Q satisfaction with outcome Rasch-transformed total scores ranged between 64.3 and 73.5 in HA_EYE_ recipients (HA_EYE_-pooled) and between 14.1 and 16.2 in the control group through Month 12 after first treatment (Figure 5). Results were generally similar in the HA_EYE_-needle and HA_EYE_-cannula groups. Among those patients receiving retreatment or their first HA_EYE_ treatment (control group) at Month 12, mean FACE-Q satisfaction with outcome scores was similar across previous HA_EYE_ recipients (72.5-72.8) and former control group patients (63.8-64.1) at Months 15 and 18.

From Month 1 through Month 12 after the first treatment, patient satisfaction questionnaire responses revealed that most patients treated with HA_EYE_ felt that they looked younger (≥71%; Month 1: 71%; Month 3: 80%; Month 12: 72%) and less tired (≥79%; Month 1: 82%; Month 3: 87%; Month 12: 79%); had reduced shadows under their eyes (≥76%; Month 1: 79%; Month 3: 83%; Month 12: 76%); and felt better about themselves (≥74%; Month 1: 81%; Month 3: 82%; Month 12: 75%). Optional retreatment (HA_EYE_-pooled) or treatment (control group) at Month 12 resulted in continued high satisfaction scores at Months 15 and 18 regarding looking younger (HA_EYE_-pooled: ≥ 84%; control: ≥ 66%) and less tired (HA_EYE_-pooled: 87%; control: ≥ 76%); and having reduced shadows under the eye (HA_EYE_-pooled: ≥ 82%; control: ≥ 79%). In the group randomized to HA_EYE_-treatment, 93% responded “Yes” at both Months 12 and 18 when asked whether they would like to receive the treatment again. Patients also reported that the treatment results looked natural throughout the study in the HA_EYE_-pooled group (90%, 92%, and 89% at Months 1, 3, and 12, and 91% and 92% at Months 15 and 18 following retreatment).

HA_EYE_-pooled needle and cannula data showed that the median time for patients to feel comfortable returning to social engagements was 2.89 hours after baseline HA_EYE_ treatment, 3.96 hours after receiving an optional touch-up treatment, and 4.70 hours after retreatment at Month 12.

### Safety Outcomes

All AESIs (associated with changes in visual functioning) were mild and not related to study product or not clinically significant. Almost all patients in both treatment groups (92% [needle], 99% [cannula]) reported 1 or more of the predefined injection-related events (bruising [67% with needle, 59% with cannula]; redness [64%, 59%]; pain [49%, 71%]; tenderness [83%, 97%]; lumps/bumps [47%, 60%]; itching [11%, 18%]; or swelling [82%, 88%]) in patient diaries after treatment at baseline, and these were typically reported to be tolerable (≥74.7%), with a duration ≤7 days (≥57%). A similar pattern also was reported following touch-up and retreatment.

In addition, investigators reported mild/moderate treatment- or procedure-related AEs for 40 (12.7%) HA_EYE_ recipients; 8 (4.9%) patients in the HA_EYE_-needle group and 32 (20.9%) in the HA_EYE_-cannula group after initial treatment (including touch-up). The most common types of treatment-related AEs (>1% of patients) were implant site swelling, implant site pain, headache, implant site bruising, implant site mass, and implant site edema (Table 5). The median time to onset of a treatment-related AE was 2 days and the median duration of AEs was 4 days. Following retreatment, 6 (3.7%) patients reported AEs related to HA_EYE_ or the procedure, all of mild intensity; 4 (2.4%) reported AEs at the site of administration and 2 (1.2%) experienced headache. Median time to onset of an AE following retreatment was 1 day and median duration of these AEs was 5 days.

**Table 5.** Treatment-related Adverse Events After HA_EYE_ Treatment: General Disorders and Administration Site (Safety Population)

	Events		
System organ class preferred term	HA_EYE_-needle *n* (%)	HA_EYE_-cannula *n* (%)	HA_EYE_-pooled *n* (%)
Initial HA_EYE_ treatment	(*n* = 163)	(*n* = 153)	(*n* = 316)
Patients with at least 1 adverse event related to study product or injection procedure	8 (4.9)	32 (20.9)	40 (12.7)
General disorders and administration site conditions	7 (4.3)	22 (14.4)	29 (9.2)
Implant site swelling	4 (2.5)	8 (5.2)	12 (3.8)
Implant site pain	0	8 (5.2)	8 (2.5)
Implant site bruising	1 (0.6)	4 (2.6)	5 (1.6)
Implant site mass	2 (1.2)	2 (1.3)	4 (1.3)
Implant site edema	0	4 (2.6)	4 (1.3)
Implant site pruritus	0	2 (1.3)	2 (0.6)
Implant site discoloration	0	1 (0.7)	1 (0.3)
Implant site induration	0	1 (0.7)	1 (0.3)
Implant site paresthesia	0	1 (0.7)	1 (0.3)
Nervous system disorders	1 (0.6)	8 (5.2)	9 (2.8)
Headache	1 (0.6)	6 (3.9)	7 (2.2)
Hypesthesia	0	1 (0.7)	1 (0.3)
Syncope	0	1 (0.7)	1 (0.3)
Skin and subcutaneous tissue disorders	1 (0.6)	3 (2.0)	4 (1.3)
Postinflammatory pigmentation change	0	1 (0.7)	1 (0.3)
Skin discoloration	1 (0.6)	0	1 (0.3)
Skin hyperpigmentation	0	1 (0.7)	1 (0.3)
Telangiectasia	0	1 (0.7)	1 (0.3)
Immune system disorders	0	1 (0.7)	1 (0.3)
Immunization reaction	0	1 (0.7)	1 (0.3)
Injury, poisoning, and procedural complications	0	1 (0.7)	1 (0.3)
Contusion	0	1 (0.7)	1 (0.3)
HA_EYE_ retreatment	(*n* = 88)	(*n* = 76)	(*n* = 164)
Patients with at least 1 adverse event related to study product or injection procedure	1 (1.1)	5 (6.6)	6 (3.7)
General disorders and administration site conditions	1 (1.1)	3 (3.9)	4 (2.4)
Implant site swelling	1 (1.1)	2 (2.6)	3 (1.8)
Implant site bruising	0	1 (1.3)	1 (0.6)
Implant site edema	0	1 (1.3)	1 (0.6)
Nervous system disorders	0	2 (2.6)	2 (1.2)
Headache	0	2 (2.6)	2 (1.2)

Initial treatment includes AEs that started on or after baseline treatment until optional retreatment. Retreatment includes AEs from patients randomized to HA_EYE_ that started on or after their optional retreatment. HA_EYE_-pooled includes all patients receiving HA_EYE_ treatment by needle or cannula. AEs, adverse events; HA_EYE_, Restylane Eyelight hyaluronic acid treatment.

## DISCUSSION

The results from this pivotal, randomized, no-treatment controlled study demonstrated that HA_EYE_ administration provides an effective, durable, and well-tolerated option for the correction of moderate and severe IOHs, regardless of the injection method (needle or cannula). Reported outcomes were consistent with those seen in previous case series studies examining the effectiveness and safety of HA_EYE_ injections in the correction of IOHs and provide further evidence of effectiveness and tolerability for this indication.^7,8,13^ Although the randomized controlled study design and study population size were overall robust for showing effectiveness and safety, one limitation was that the study population consisted mainly of White (89%) and female (87%) patients and therefore did not represent all ethnicities or both sexes to equal extent.

The pivotal randomized study met the primary endpoint. HA_EYE_ treatment resulted in a higher GIHS responder rate when compared with the no-treatment control group at Month 3 after the first treatment, and the between-group difference was statistically significant (*P* < .001). HA_EYE_ treatment effectiveness was durable and sustained throughout the study period, with the between-group difference in GIHS responder rate remaining statistically significant throughout the study period up to Month 12. (*P* < .001). This translated to a duration of 11 to 12 months after the last treatment, depending on whether patients received a touch-up treatment or not at Month 1. These results compare favorably with systematic review data from 8 published studies, which indicate that duration of treatment effect typically lasts for 10.8 months.^7,19^ Retreatment at Month 12 restored GIHS responder rate alongside GAIS and FACE-Q satisfaction scores, all of which remained high through Month 18. Further studies are needed to evaluate durations beyond 12 months without retreatment.

Investigator-assessed GAIS scores were above 87.5% in HA_EYE_ recipients, highlighting the overall aesthetic results observed with IOH correction and tear trough rejuvenation, and remained similar throughout the study, irrespective of whether treatment was administered by needle or cannula. Patient-assessed GAIS responder rates were also similar to investigator-reported data. Systematic review data suggest that HA fillers provide aesthetic improvements for 6 to 12 months; the outcomes reported in the current study are therefore consistent with the longest durations published.^19^ These results are in line with other published studies, which typically show sustained and high levels of aesthetic improvement with HA fillers for correction of IOHs.

Patient satisfaction is an important indicator of treatment success and also a key factor in decisions to undergo further cosmetic procedures.^20^ Patient perspective when evaluating treatment effectiveness, as well as tolerability and safety aspects, should be included in studies. Patient-reported satisfaction is typically high in studies in which correction of tear trough abnormalities with HA filler products is investigated.^19,21-23^ Our results reflected those previously reported for HA fillers, with elevated levels of satisfaction after HA_EYE_ treatment.^19,21-23^ High FACE-Q satisfaction with outcome Rasch-transformed total scores showed that HA_EYE_ recipients were satisfied with their treatment from Month 1 through Month 12 after first treatment (mean range: 64.3 to 73.5) compared with the no-treatment control group (mean range: 14.1 to 16.2). Patients who received retreatment at Month 12 continued to report high satisfaction with HA_EYE_ treatment through Month 18. Again, results were generally consistent between patients in the HA_EYE_-needle and HA_EYE_-cannula groups and reflected previous FACE-Q data associated with HA filler treatment in the tear trough area.^7,23^ HA_EYE_ treatment was associated with high patient satisfaction questionnaire scores, with the majority indicating that they looked less tired (>78%) and younger (>70%), with reduced shadows under their eyes (>76%). Recovery time was rapid following HA_EYE_ administration, with participants feeling comfortable to return to social engagement within 3 hours after baseline treatment and approximately 4 to 5 hours after touch-up or retreatment.

HA_EYE_ treatment was generally well tolerated throughout the study, with typically mild treatment-related AEs reported, which occurred around the eye area (eg, edema, bruising) and were aligned with those reported in previous HA_EYE_ studies.^7,8,13^ Visual function was unaffected by HA_EYE_ injections and no AESIs were considered related to the study treatment. There were no confirmed cases of Tyndall effect in the current study, whereas Tyndall effect (bluish discoloration) has been reported following initial and repeat treatments with other HA filler products.^22^

The data reported here demonstrate that HA_EYE_ injections provide an effective, durable, and well tolerated nonsurgical option for the correction of IOHs. Rapid recovery time and high response rates following retreatment (with typically mild complications reported) make HA_EYE_ a convenient option that can be offered as a repeat treatment for corrections of abnormalities in this challenging facial area.

## CONCLUSIONS

This pivotal study demonstrated HA_EYE_ to be effective and well tolerated for the correction of moderate to severe IOHs in patients over 21 years of age. The IOH correction was sustained in the majority of patients through Month 12 after the first treatment and up to Month 18 for individuals retreated at 12 months. GIHS improvement was similar regardless of whether treatment was performed with a needle or cannula. Aesthetic improvement (GAIS) and patient satisfaction (FACE-Q) were high throughout the study, and the treatment was well tolerated.
